# Illustrated Manual of Operative Surgery and Surgical Anatomy

**Published:** 1852-04

**Authors:** 


### BIBLIOGRAPHICAL NOTICES.

---

*Illustrated Manual of Operative Surgery and Surgical Anatomy*, By MM. Cl. Bernard and Ch. Huette; *edited with notes and additions, and adapted to the use of the American Medical Student*, by W. H. Van Buren, M. D., Surgeon to Bellevue Hospital, &c., and C. E. Isaacs, M. D., Demonstrator of Anatomy College of Physicians and Surgeons, New York. *Illustrated with steel engravings from drawings after nature*, by M. J. Léveillé, *designed to serve as a companion to the ordinary text books of Surgery*. New York, H. Baillière, 290 Broadway, 1852. Part I., pp. 84, and 26 plates with 130 figures.

The rather lengthy title here presented will afford our readers a good idea of the very handsome book which is thus offered to their notice. We need say nothing in commendation of the French original. Its reputation as a convenient and beautiful elementary atlas of operative surgery, has long been established; and many of the plates have recently become still more widely known, through the excellent American copies of them in the published parts of the operative surgery of Dr. H. H. Smith. The translators are men of tried ability and fitness for their undertaking, and we doubt not will acquit themselves in the very best manner of the task before them. This they appear to have done fully in the part now issued; but we hope to be able to speak more at large and decidedly in this connexion, after the remainder of the volume has arrived. In the mean time it may be well to note a few matters of interest in regard to the character and contents of the part before us, as well as to the views and intentions of the American editors, according to the language of their preface.

These gentlemen "flatter themselves that they have contributed to the supply of a want which has not been unfrequently experienced heretofore, viz: a complete and concise picture of the science and art of operative surgery, in its present advanced and perfected condition, in a portable form. The admirable and extensive works of Bourgery and Jacob, and Velpeau with the translation of the latter under the auspices of Prof. Mott, and the equally excellent treatise on operative surgery by Prof. Pancoast of Philadelphia, can never be replaced by the present work. Yet its compactness and portability will render it more desirable to the student as a companion in the lecture and dissecting room, where its copious and graphic illustrations will assist him materially in acquiring correct general ideas as to the nature and objects of the individual operations of surgery; whilst for more minute and varied details with regard to their history and numerous modifications, the less accessible and more expensive treatises alluded to, can be consulted at a more advanced period of study. In fact, they are better calculated for works of reference to the practitioner of surgery, than as text books for the student, designed to set forth concisely the elements of the art. Our manual, whilst it is intended mainly to illustrate the intricacies of operative surgery, by appealing to the eye as well as to the understanding of the student, and by familiarizing him with that most useful department of anatomy which relates to surgical operations, will also be found, it is hoped, not entirely useless as a work of reference to those already engaged in practice."

We are glad to learn further, that in the fulfilment of their plan the translators have especially endeavored "to Americanize the language of the work to as great an extent as possible, making use of the terms in ordinary use in this country by teachers of anatomy and surgery, in order that the American student may not be annoyed by meeting with foreign modes of expression with which he is not familiar, and which, in their opinion, it is rarely desirable to introduce into common use."

The first six plates are devoted to the representation in some forty-eight figures of the "Instruments employed in Surgical operations." These, as a matter of course, are all French, and after the patterns of Charriére. Among them we have, 1st, instruments required for making incisions; 2d, those required for ligature of arteries: 3d and 4th, those required for amputations; 5th and 6th, for exsection of bones. Next in order come the operations themselves; and here again we have six preliminary plates occupied, this time, with various minor or elementary manipulations, such as incisions, union of wounds, the employment of the seton, vaccination, scarifications, acupuncture, the anatomical and operative surgery of bleeding in different parts, including arteriotomy; and lastly, the ligature of arteries in general.

After these are exhibited, an admirable series of illustrations of the surgical anatomy and operative surgery, side by side, of the ligature of arteries in particular, begining in plate vii., with the surgical anatomy and ligature of the ulnar and radial arteries, and ending in plate xvii., with the anatomy and ligature of the femoral artery under Poupart's ligament, of the external iliac and epigastric arteries.

The remaining eight plates are occupied with the several amputations through the joints, or disarticulations. These engravings, being duplicates of the Parisian originals, we need hardly say are splendidly executed and printed, and in every way adapted to the purpose for which they were designed. The letter press accompaniments are brief but concise and clear, and appear to be rendered into English faithfully and elegantly, without any sacrifice of perspicuity. They afford all the assistance that would be looked for in a work that is manifestly one of graphic demonstration and illustration, rather than of written description. The annotations of the Editors, which are very modestly appended, seem to be judicious and appropriate; and thus far likely to increase the value of the book to the American reader. We cannot take leave of them, however, without a word or two in relation to the foot-note on pages 19 and 20, in which, what *we* call the Philadelphia needle, is spoken of as the artery-needle, "which bears the name of Prof. Mott," and is "also known as the American needle." Now, although we have no violent objection to its designation as the American needle, we decidedly protest against its being called after its distinguished New York advocate and endorser. It is well known here that Prof. Mott had nothing whatever to do with the contrivance of the instrument in question, or with its first employment and description. The merit, such as it is, belongs to the late Dr. Joseph Parrish, of this city, who published a full account and engraving of it in the Eclectic Repertory, many years ago, (vol. 3, p. 229, Philadelphia, 1813.) Wherein he expressly and repeatedly attributes its invention to Dr. J. Hartshorne and himself, aided by the ingenuity of Mr. John Rorer, of this city.

We regret that the editors did not happen to refer to the additional article on aneurisms in the New York translation of Velpeau's Operative Medicine. The note at the foot of page 302, (vol. 2,) would have at once reminded them that Prof. Mott distinctly admits his favorite needle to have been the joint production of three Surgeons of Philadelphia, Drs. Parrish, Hewson and Hartshorne. Surely if a special name were needed, here was choice enough without wandering quite so far from home. The mere question of name is a matter of small moment practically, beyond a doubt; still it is a matter of history, in which is involved a principle not less important in small things than it is in great —we mean the principle of *suum cique.*

